# Supplementary material for: Moderate greenhouse climate and rapid carbonate formation after Marinoan snowball Earth
Source: Nat Commun. 2024 Apr 26;15:3571. doi: 10.1038/s41467-024-47873-6 (PMC11053170; doi:10.1038/s41467-024-47873-6)
Supplement: Supplementary file 1 — Supplementary information [file 41467_2024_47873_MOESM1_ESM.pdf]

Supplementary Information for:
Moderate greenhouse climate and rapid carbonate formation after Marinoan snowball Earth

Authors: Lennart Ramme[1,2*], Tatiana Ilyina[3,4,1], Jochem Marotzke[1,3]

[1] Max Planck Institute for Meteorology, Hamburg, Germany
[2] International Max Planck Research School on Earth System Modelling, Hamburg, Germany
[3] Center for Earth System Research and Sustainability (CEN) - Universität Hamburg, Hamburg, Germany
[4] Helmholtz-Zentrum Hereon, Geesthacht, Germany
[*] corresponding author (lennart.ramme@mpimet.mpg.de)

List of Tables

S1 Overview table of the ICON-ESM simulations . . . 2

List of Figures

S1 Time series of global variables from the ICON-ESM simulations, part 1 . . . 3
S2 Time series of global variables from the ICON-ESM simulations, part 2 . . . 4
S3 Impact of the dilution effect on the carbon cycle as calculated with a box model . . . 5
S4 Effect of suppressed primary production on the carbon cycle . . . 5
S5 History of prescribed CO_2 emissions and atmospheric CO_2 concentration . . . 6
S6 Evolution of global ice coverage and mean 2m air temperature . . . 6

Table S1: Overview table of the ICON-ESM simulations. Presented are the physical and chemical conditions at the start of the deglaciation, which either result from the preceding modelling procedure or were set manually (italic font). The concentrations can be represented by a global value, because the ocean is well mixed below the ice of a snowball Earth. The values in the second part of the table show the conditions after 5000 simulation years. A description of the experiments is given in the Method section.

	Exp. 1	Exp. 2	Exp. 3.1	Exp. 3.2	Exp. 3.3	Exp. 4	Exp. 5
**Conditions at start of deglaciation**							
- atm. CO_2 (ppm)	13710	13710	13710	13710	13710	13720	13710
- ice thickness (m)	147	147	147	147	147	149	147
- ocean salinity (psu)	35.5	35.5	35.5	35.5	35.5	50.5	35.5
- ocean temperature (°C)	-1.8	-1.8	-1.8	-1.8	-1.8	-1.8	-1.8
- TA (mol m^{-3})	2.47	*6*	*15*	*15*	*15*	*15*	*15*
- DIC (mol m^{-3})	2.12	*5.05*	*15.75*	*15.75*	*15.75*	*15.75*	*14.1*
- PO_4 (mol m^{-3})	0.0026	10^{-2}	10^{-2}	10^{-2}	10^{-4}	10^{-2}	10^{-2}
- NO_3 (mol m^{-3})	0.025	0.025	0.025	0.025	0.025	0.036	0.025
- O_2 (mol m^{-3})	0.16	10^{-3}	10^{-3}	10^{-3}	10^{-5}	10^{-3}	10^{-3}
- Fe (mol m^{-3})	10^{-6}	$5 \cdot 10^{-6}$	$5 \cdot 10^{-6}$	$5 \cdot 10^{-6}$	$5 \cdot 10^{-6}$	$5 \cdot 10^{-6}$	$5 \cdot 10^{-6}$
- ocean pCO_2 (ppm)	115	178	13329	13329	13202	15086	1462
- mean surface pH	8.5	8.7	7.3	7.3	7.3	7.3	8.2
- Ω_{CaCO3} (below ice)	5.4	18.9	3.3	3.3	3.4	2.5	24.1
**Conditions after 5000 simulation years**							
- atm. CO_2 (pppm)	7969	4319	14681	17492	17192	12993	11728
- GSAT (°C)	23.3	17.6	29.3	31.4	31.4	27.5	26.5
- GSAT Drift (K (100 yr)$^{-1}$)	±0.0	±0.0	+0.05	+0.1	+0.07	+0.05	+0.07
- mean surface pH	6.68	7.36	7.30	7.20	7.22	7.19	7.31
- total integrated carbonate formation (10^{15} mol)	0.0	0.01	-	691	226	-	1532
- NPP (phyto.) (GT C yr^{-1})	38	55	63	62	7.6	58	61
- NPP (cyano.) (GT C yr^{-1})	32	81	102	107	0	75	96

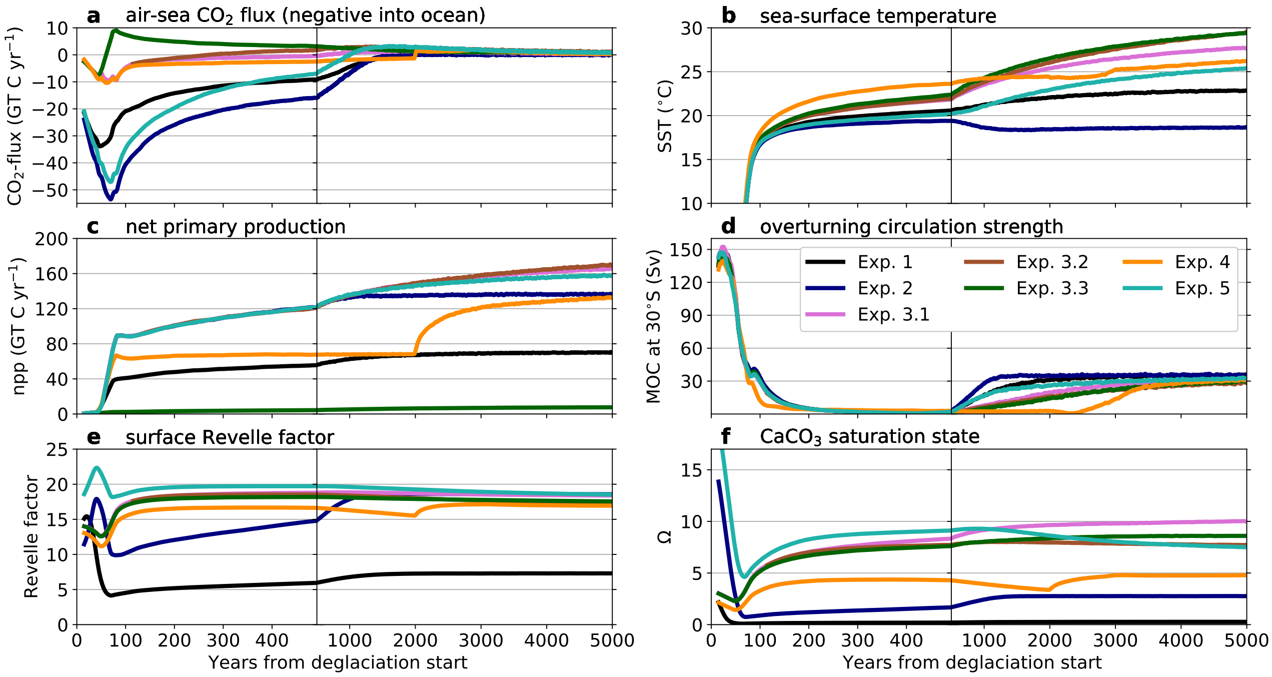

Figure S1: Time series of global variables from the ICON-ESM simulations, part 1. For more information on the experiment settings see Fig. 1 in the main article file and the list of experiments in the Method section. All time series show 30-year running mean data. Note the change in scale of the time axis at year 500. In year 2000 of Exp. 4 the surface freshwater input is stopped, causing the kink of the time series at that point. **a**, Annual air-sea CO_2-flux, note the change in scale of the y-axis, **b**, mean sea-surface temperature, **c**, sum of global annual primary production by phytoplankton and cyanobacteria, **d**, strength of meridionally averaged overturning circulation at the location of its maximum in the control simulation, **e**, mean surface Revelle sensitivity factor, **f**, mean surface calcium carbonate saturation state.

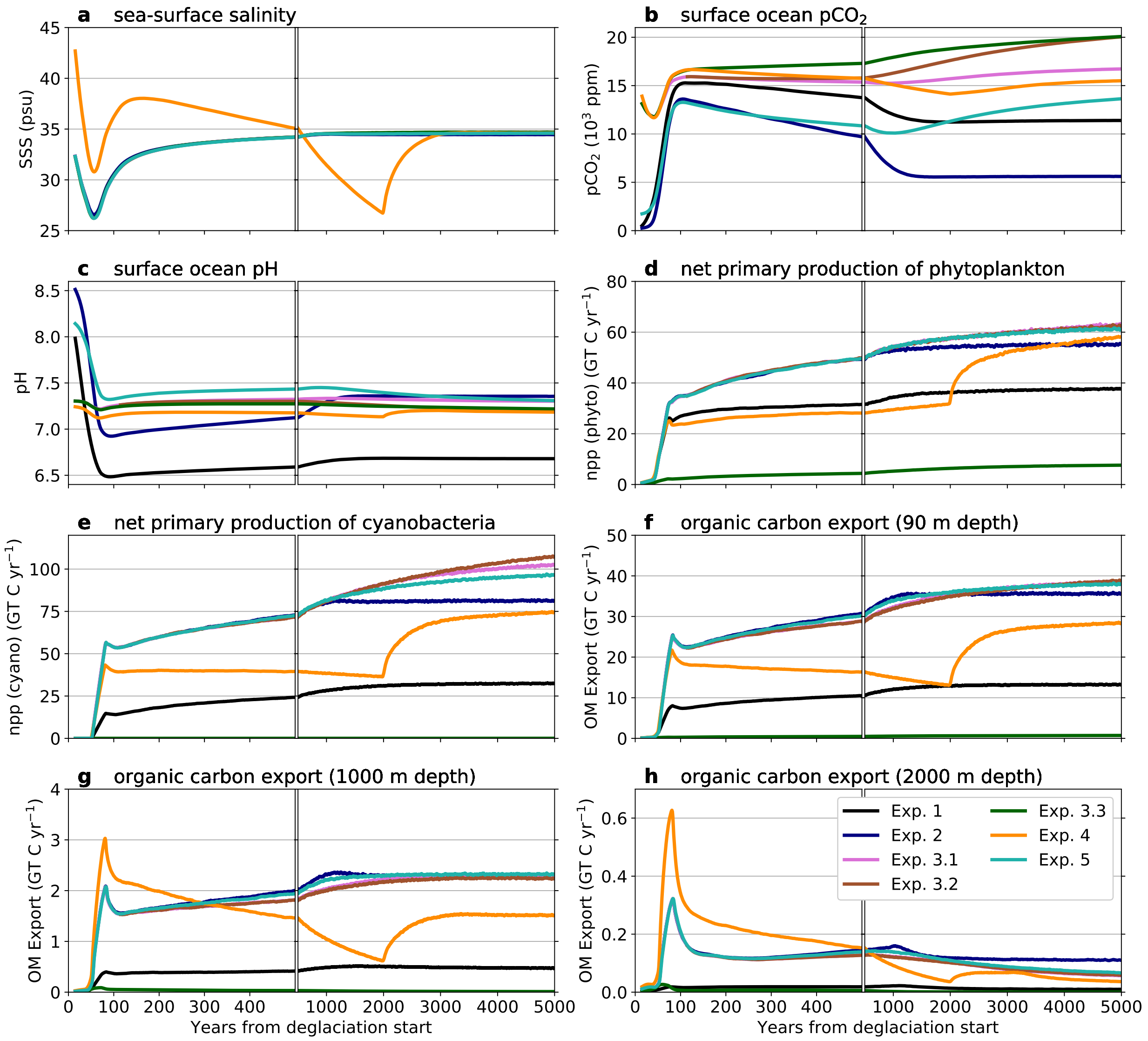

Figure S2: Time series of global variables from the ICON-ESM simulations, part 2. See Fig. S1 for more general information. **a**, mean sea-surface salinity, **b**, mean surface ocean pCO_2, **c**, mean surface ocean pH, **d**, total global primary production of phytoplankton, **e**, total global primary production of cyanobacteria, **f-h**, total global downward organic matter transport at depths of 90, 1000 and 2000 m.

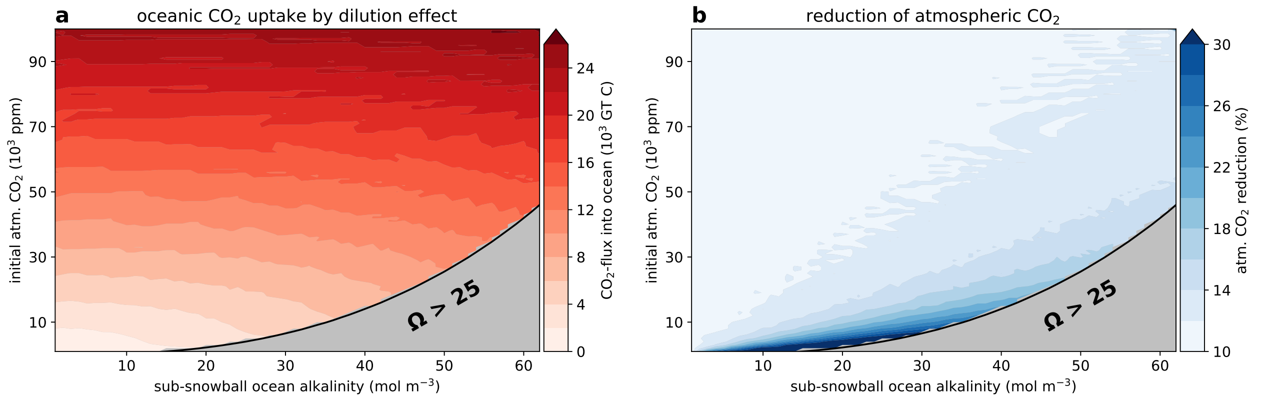

Figure S3: Impact of the dilution effect on the carbon cycle as calculated with a box model. Shown are the oceanic carbon uptake (**a**) and the corresponding relative reduction of the atmospheric CO_2 concentration (**b**). The isolated effect is calculated for a scenario in which meltwater with a volume of 1000 m of sea-level equivalent is instantaneously mixed with the sub-snowball ocean, as described in the main text. The conditions in the lower right corner are not sustainable during a snowball Earth, because the carbonate saturation state would be too large.

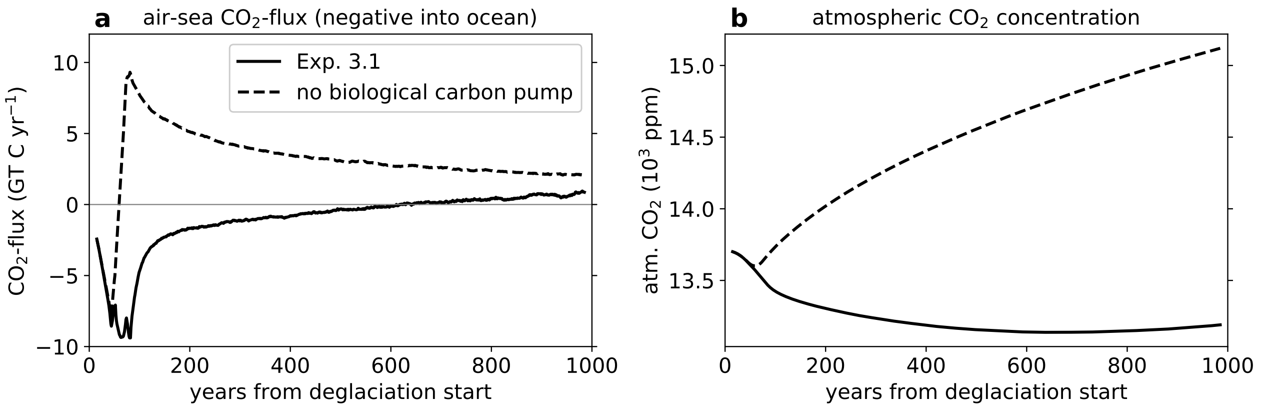

Figure S4: Effect of suppressed primary production on the carbon cycle. Shown are the air-sea CO_2-flux (**a**) and the atmospheric CO_2 concentration (**b**) of Exp. 3.1 and a test simulation with suppressed primary production. The chosen time period represents the main phase of the deglaciation, where both temperature and primary production increase strongly. The data is represented by 30-year running means.

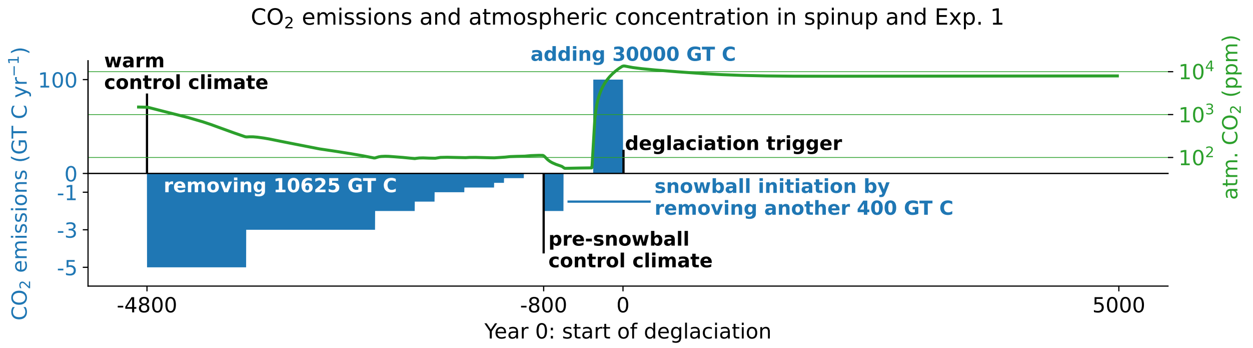

Figure S5: History of prescribed CO_2 emissions and atmospheric CO_2 concentration. From year 0 on, the data of Exp. 1 is shown. See Fig. 1 in the main article file for time series of the CO_2 concentration in the other experiments. The simulation procedure is described in the Method section.

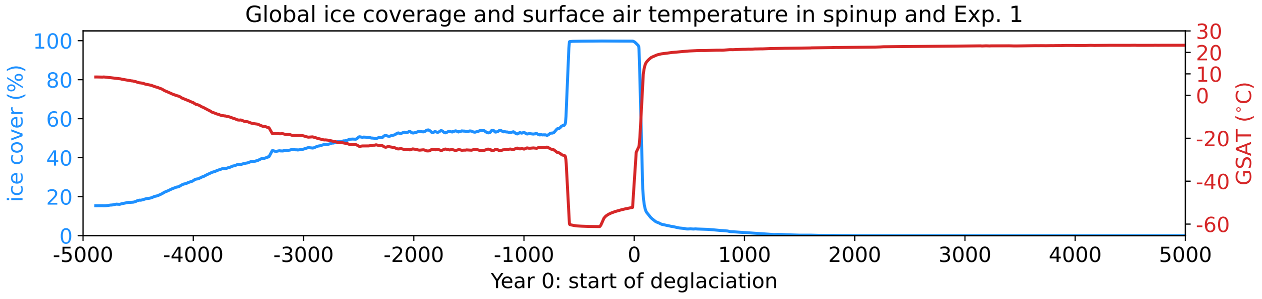

Figure S6: Evolution of global ice coverage and mean 2m air temperature. The small kink in the time series around year -3300 is coming from a modification made in the formulation of sea-ice dynamics in the model.
